# Disease behaviours of sows naturally infected with *Taenia solium* in Tanzania

**DOI:** 10.1016/j.vetpar.2017.01.008

**Published:** 2017-02-15

**Authors:** Chiara Trevisan, Maria Vang Johansen, Ernatus Martin Mkupasi, Helena Aminel Ngowi, Björn Forkman

**Affiliations:** a Department of Veterinary and Animal Sciences, University of Copenhagen, Dyrlægevej 100, 1870 Frederiksberg C, Denmark; b Department of Veterinary Medicine and Public Health, Sokoine University of Agriculture, P.O. Box 3021, Morogoro, Tanzania; c Department of Veterinary and Animal Sciences, University of Copenhagen, Grønnegårdsvej 8, 1870 Frederiksberg C, Denmark

**Keywords:** *Taenia solium* cysticercosis, Sickness behaviour, Animal welfare, Pigs, Burden of disease, Sub-Saharan Africa

## Abstract

Neurocysticercosis (NCC) is a disease caused by the zoonotic parasite *Taenia solium* lodging in the central nervous system. Both humans and pigs can get NCC. The impact of the disease in pigs has so far been little explored. The aim of this study was to describe the effect of NCC on social and feeding behaviours as well as the pattern of activity as indicators of reduced welfare in naturally infected sows.

In total 13 *T. solium* naturally infected and 15 non-infected control sows were videotaped for 2 consecutive weeks using close circuit television cameras at research facilities at Sokoine University of Agriculture, Morogoro, Tanzania. Videos were analysed at the beginning, in the middle and at the end of the 2 week recording period. For each time point, videos were analysed during feeding, while the enrichment was provided, and by recording every half an hour the sows’ behaviours performed over the course of a whole day.

Sows with NCC spent significantly less time at the feeding trough, especially during the second half of the feeding period. Infected sows were also more passive e.g. lying and standing still significantly more during a whole day period and showed social isolation compared to non-infected control sows by performing behaviours more distant to their nearest neighbour. Results of this study indicated that NCC changed the behaviour of infected sows. The behavioural changes are indicative of decreased welfare. Efforts to reinforce the animal welfare aspect are needed as this has so far been neglected.

## Highlights

• Infected sows spent significantly less time at the feeding trough.
• Infected sows were also more passive by lying and standing still significantly more during a whole day period.
• Infected sows showed social isolation by performing behaviours more distant to their nearest neighbour.
• Neurocysticercosis changed the behaviour of infected sows.
• The observed behavioural changes are indicative of decreased welfare.

## Graphical abstract

**Figure:**
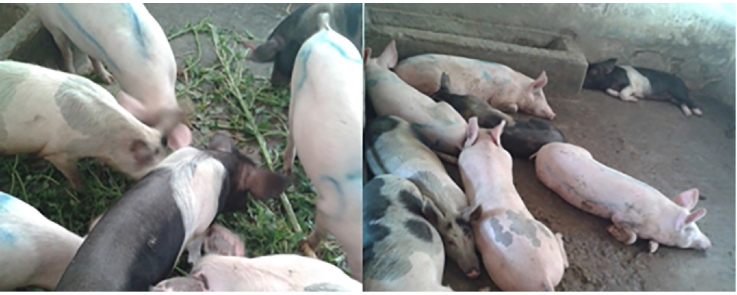

## 1 Introduction

Neurocysticercosis (NCC) is a disease caused by the zoonotic parasite *Taenia solium* lodging in the central nervous system. Both humans and pigs can get NCC (Garcia et al., 2003). In pigs the disease has long been considered a solely economic problem as infected pork is either condemned or sold at a reduced price (Phiri et al., 2003, Trevisan et al., 2017).

Most definitions of animal welfare include freedom from disease as an important part of animal welfare it is e.g. one of FAWC’s five freedoms (Farm Animal Welfare Council 1992) and one of the criteria of the Welfare Quality^®^ protocols (Botreau et al., 2007). The degree to which a disease affects the behaviour of an animal may indicate the welfare consequence of that disease (FAWC, 2012).

Various examples of sickness behaviours and mechanisms of survival have been reported in literature (Weary et al., 2009). Ill animals show limited interest in drinking and eating, are frequently described as lethargic and depressed and often present with rough hair coats, indicating a lack of grooming (Hart, 1988). More recent studies have shown that particular behaviours (e.g. hypersomnia, decreased activity) are performed as adaptive behaviours to save energy and resources that can be allocated to fight the pathogen or disease (Dantzer, 2004). This may thus indicate that the behaviour of a sick animal is a functional response to illness (Hart, 1988).

The effect of parasitic diseases on pig behaviour and welfare has not been studied in great detail. Damm et al. (2003) carried out a study on the gradual weaning process in relation to nematode infections in outdoor sows and piglets. The authors could show that sows’ infection status had some effects on sow and piglet activities between nursing, but could not reject their hypothesis that nematode infection would affect gradual weaning. Mejer et al. (1999) observed that parasitic worm infections affect piglets’ growth due to changes in the intestine, but previously reported negative effects of helminths on sow reproductive performance could not be confirmed.

Whilst in humans the impact of NCC has been well studied and described, there is little information on the impact of NCC in pigs (Mkupasi et al., 2014). Only a limited number of studies have assessed the effects of the parasite on the pig, the natural intermediate host for *T. solium* (Prasad et al., 2006, Mkupasi et al., 2014, Trevisan et al., 2016).

The aim of this study was to describe the effect of NCC on social and feeding behaviours as well as the pattern of activity as indicators of reduced welfare in naturally infected sows.

## 2 Materials and methods

### 2.1 Animals

For the study 28 sows, 13 naturally infected with *T. solium* and 15 non-infected control sows were included. Infected sows were bought in Dodoma region, a rural area where the disease is prevalent as pigs are left free roaming and sanitation and hygiene systems are inappropriate (Mkupasi et al., 2014). Infection was confirmed by lingual examination and the inclusion criterion was sows with more than three lingual cysts (Dorny et al., 2004). The non-infected pigs were purchased in Morogoro region, an urban area where porcine cysticercosis is absent as pigs are raised in semi commercial farms without access to free roaming (Makundi, 2012). Exclusion criteria were sows: younger than 6 months, less than 50 cm in height, under two thirds of the average weight of 40 kg for a healthy adult Tanzanian pig, heavily infested with ectoparasites and/or with injuries. Non-infected control sows were matched subsequently with infected ones. On arrival at Sokoine University of Agriculture (SUA), all sows were clinically examined. To eliminate possible confounders (endoparasites and ectoparasites) a subcutaneous injection of 0.3 mg/kg of ivermectin (ivermectin ALFAMEC^®^ 1% Batch No. 1305136–01) was administered to all animals twice, at an interval of two weeks (Barragry, 1987).

### 2.2 Experimental design

The study was carried out at the experimental animal facilities at SUA, Morogoro, Tanzania. A parallel group design was formed. As the disease cannot spread between pigs infected sows and respective non-infected controls could be housed in the same stable. In total four pens were formed. Pen 1 and 4 contained three infected and three non-infected control sows, pen 2 contained three infected and five non-infected control sows and pen 3 contained four infected and four non-infected control sows (see Fig. 1 in Supplementary material). Moreover, pen 1 and 4 housed larger sows, while in pen 2 and 3 the sows were of smaller sizes.

Each pen measured 4 × 3 m with a cement floor and walls and a cement trough (1.5 × 0.5 m). The pens were cleaned daily and the sows were fed twice a day with commercial dry pig feed. Water was provided ad libitum and after morning feeding the animals were provided enrichment consisting of leaves and branches of *Leucaena leucocephala* and *Amaranthus spinosus*. The mean room temperature of the stable was of 25 °C. Light was present from 6:00 h until 19:00 h. The sows were kept at the experimental animal facilities for 1 month (2 weeks of acclimatization and 2 weeks of video recording).

The sows were weighed using a beam balance at arrival and then at intervals of 9 days until all the animals were sacrificed. For results of cysticerci distribution see Table 1 in Supplementary material (Trevisan et al., 2016).

### 2.3 Video recording and data collection

During two weeks of video recording, sows were continuously videotaped using close circuit television (CCTV) cameras (Velleman^®−^ CCTVPROM16). One CCTV camera was mounted above each of the four pens in central position, permitting a top view of the whole pen. Each sow was colour-marked on its back and on its sides using coloured stock markers to allow individual identification on the video recordings.

Videos were analysed at the beginning (8th November 2014), in the middle (14th November 2014) and at the end (20th November 2014) of the 2 week recording period (see Fig. 1 in Supplementary material). For each time point, videos were analysed using one minute scan sampling during feeding, defined as the period when the fodder was added to the feeding trough until it was finished and the enrichment was provided. The enrichment period was also studied using one minute scan sampling, defined as the period when the enrichment was added to the pen until it was consumed by the sows. Finally by scan sampling every half hour from 00:00 h to 23:59 h of one day (Martin and Bateson, 1993).

### 2.4 Behavioural observations and hypotheses

Individual behaviour patterns were defined as behaviour patterns performed by a single animal (Jensen, 1980).

#### 2.4.1 Feeding period

Our hypothesis was that during feeding infected sows would spend less time at feeding trough compared to non-infected control sows.

Every minute it was recorded which sows were present at the feeding trough defined by having at least the head inside the trough.

To estimate the frequency of feeding trough presence the feeding period was divided into two intervals of equal length (“First feeding half” and “Second feeding half”) (interactions/feeding interval) (Mendl et al., 1992).

#### 2.4.2 Enrichment period

Our hypothesis was that infected sows would show less interest and spend less time interacting with the enrichment than non-infected control sows.

The following mutually exclusive states were recorded, namely (1) interaction with enrichment: Sow in contact with the enrichment, leaves and branches of *L. leucocephala* and *A. spinosus*, (2) active behaviour not towards enrichment: Sow in contact with the feeding trough, scratching, drinking, (3) feeding: Sow having the head inside the trough, (4) standing: Sow standing without doing anything and (5) lying down: Sow lying on its sternum or on either of its sides without doing anything.

Every minute in which the sow was interacting with the enrichment was recorded during the period where enrichment was given.

For data analysis the enrichment period was divided into five intervals of equal length (“a”, “b”, “c”, “d” and “e”) from the beginning to the end of the enrichment period.

#### 2.4.3 Whole day

Our hypothesis was that during the whole day period infected sows would be more passive (lying or standing) than the non-infected control sows and also show social isolation by lying in greater isolation compared to non-infected control sows.

In addition the distance to nearest neighbour was noted by recording every half an hour how far the sows were to each other using 4 scores, namely (1) Score 1: sow is in contact with the other sow, (2) Score 2: one sow’s head size from each other, (3) Score 3: one sow’s length from each other and (4) Score 4: more than one sow’s length from each other.

For data analysis the whole day period (from 00:00 h to 23:59 h) was divided into 5 time intervals, namely night 1, before feeding, after feeding (morning), after feeding (afternoon) and night 2.

Active behaviours such as: (1) interaction with enrichment, (2) active behaviour not towards enrichment, (3) feeding and passive behaviours such as: (4) standing, (5) lying down, were summed, respectively.

### 2.5 Statistical analysis

The frequency of the behavioural observations and sows weight and id was analysed as continuous variables, while infection status, sampling day and sow size (large, small) were analysed as dichotomous variables. Data were analysed using the Mann-Whitney *U* test, the Kruskal-Wallis test and repeated Linear Mixed Effects models. Infection status (yes/no), sampling day (1, 2, 3) and weight (large, small) were included as fixed effects while sow id (1–28) and pen (1–4) were included as random effects. Interactions were only included if significant. Results were considered significant when p-values were ≤0.05. Data were analysed using the software environment for statistical computing and graphics R version 3.2.2 (R Core Team, 2016).

### 2.6 Ethical approval

All procedures employed in the study were approved by SUA, Morogoro, Tanzania (Ref. no. RPGS/R/AS/42/2014). The study was performed in accordance to the national guidelines of ethics for health research and to the Tanzanian animal welfare act (2008) (The United Republic of Tanzania, 2008, Mashalla et al., 2009).

## 3 Results

No significant difference was observed in weight gain in the 4 pens (Pen1: p = 0.45, Pen2: p = 0.39, Pen3: p = 0.44, Pen4: p = 0.49), therefore the mean weight was calculated for each sow. A significant difference between the pens of large compared to smaller sows existed (p = 0.0002). No weight difference was observed between infected and non-infected control sows (p = 0.26).

**Fig. 1:**
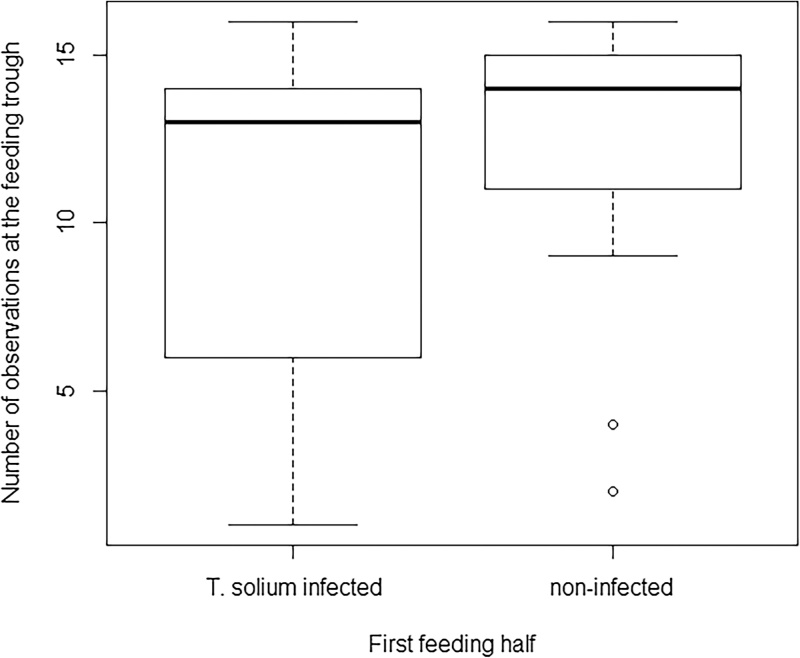
Number of observations at the feeding trough for *T. solium* infected and non-infected control sows during the first feeding half.

**Fig. 2:**
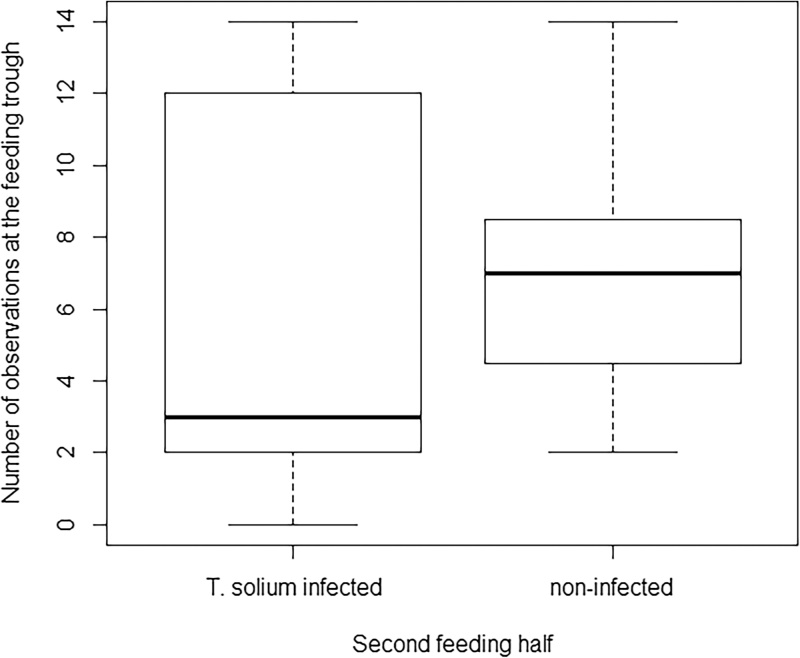
Number of observations at the feeding trough for *T. solium* infected and non-infected control sows during the second feeding half.

The number of observations at the feeding trough was significantly lower for infected sows in the second half of the feeding period (p < 0.0001). The null hypothesis could be rejected as infected sows spent less time at feeding trough compared to controls (Figs. 1 and 2 ).

**Fig. 3:**
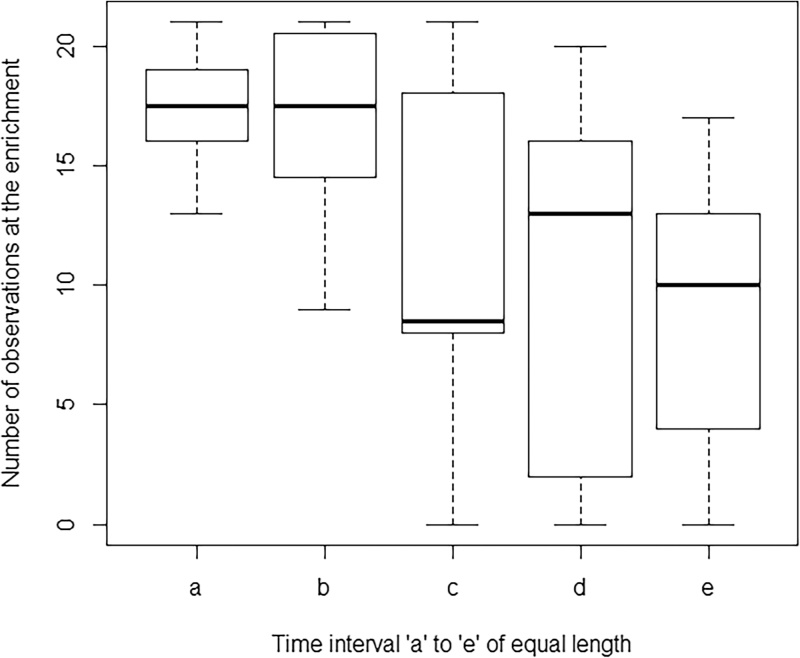
Number of *T. solium* infected and non-infected control sows’ contacts with the enrichment per time interval a–e.

Significant differences were observed between enrichment time intervals “a”–“e”, where with increased time significant fewer contacts with the enrichment were observed (Fig. 3). However, no difference was observed between infected and non-infected control sows in time spent interacting with the enrichment (p = 0.35). The null-hypothesis could therefore not be rejected.

A significant difference between infected and non-infected control sows was observed in frequency of active and passive behaviours (p = 0.0049). Infected sows showed to be more passive and less active than non-infected control sows over a period of a whole day. In addition, the animals’ weight also seemed to affect the passive behaviour, where larger sows tended to spend more time performing passive behaviours compared to smaller sows (p = 0.0007).

Moreover, infected sows performed behaviours more distant from their nearest neighbour compared to non-infected control sows (p = 0.0347). The null hypothesis could be rejected as infected sows were more passive by lying and standing still more compared to non-infected control sows and showed social isolation by lying in greater isolation compared to non-infected control sows.

## 4 Discussion

The results of the study indicated that NCC changed the behaviour of infected sows. Sows with NCC spent less time at the feeding trough, were more passive and showed social isolation compared to non-infected control sows. Taken together these changes in behaviour indicate that NCC leads to a decreased welfare.

While fodder was abundant, infected and non-infected control sows were equally present at the feeding trough; however during the second interval of the feeding period sows with NCC spent significantly less time at feeding trough compared to non-infected control sows. These results indicate that when the energy requirement for obtaining fodder was greater as fodder was getting less abundant overtime; infected pigs seemed to perform less energy intensive behaviours such as sleeping or standing still (Aubert, 1999). The latter was also observed in a study where endotoxin injected rats stopped pressing the bar to obtain water, but drank it when water was provided (Miller, 1964) and further supported by a study of interleukin-1 treated rats, who stopped pressing a lever for food but still ate the food pellets which were delivered independently of their behaviour (Aubert et al., 1995). In a study on cattle feeding time decreased and remained low only a few hours after *Escherichia coli* inoculation suggesting that even when feed was easily accessible, illness affected the feeding behaviour (Fogsgaard et al., 2012).

When we observed the sows’ behaviour during the enrichment period, infection seemed not to play a role as no difference in time spent interacting with the enrichment was observed between infected and non-infected control sows. This might be explained by the fact that the enrichment consisted of food plants (*A. spinosus* or braches of *L. leucocephala* with leaves) which was distributed in abundance all over the pen; hence no competition was necessary and all sows could forage undisturbed. This is in accordance with Hart (1988) who reported that when food was easily accessible it was not uncommon in domestic animals to see a willingness to take food even during illness (Hart, 1988). The fact that the animals did not need energy to forage or hunt for the food could further explain the lack of difference between the two groups (Johnson, 2002).

Infected sows showed to be more passive than non-infected control sows when the sows’ behaviour was assessed over a whole day. Sows with NCC spent more time lying and standing still compared to non-infected control sows. These results are in line with Damm et al. (2003), who showed that nematode infected sows spent more time in the huts (42% versus 29% of scans, p = 0.02) compared to healthy controls. In many instances of infection animals adopt the energy conservation strategy which consists in reducing activity and increasing sleepiness (Aubert, 1999). In this study it appeared that sows performed behaviours that mirrored those of sick animals potentially conserving energy to fight infection (Johnson, 2002). These results support the hypothesis underlined in some recent research that sickness behaviour is a well-organized adaptive response by the host designed to facilitate recovery and enhance disease resistance in periods critical for the survival of the animal (Johnson, 2002).

Finally in this study the passive behaviours of NCC sows were also performed more distant from their nearest neighbour. These are also behaviours that mirror those of a sick animal that often are described as lethargic, isolated and uninterested (Hart, 1988). In humans the clinical signs of NCC can be varied and non-specific, however epileptic seizures account for the majority (Garcia et al., 2003). Seizures were also observed in two pigs of this study (Trevisan et al., 2016), and histopathological findings (Christensen et al., 2016) suggested that collagen was likely to play a considerable role in the pathogenesis of seizures in NCC. It needs to be underlined that in human NCC the majority of patients are asymptomatic, phenomenon that was also noticed in the pigs in Trevisan et al. (2016).

Sows in this study showed social isolation. The greater distance between infected and non-infected control sows indicated a form of lack of welfare. The latter, can be measured in humans using quality of life assessments (de Almeida and Gurjao, 2010). Results from a study performed by de Almeida and Gurjao (2011) suggested that patients with NCC had a worse quality of life compared to the group without NCC as the disease affected the emotional, physical and social aspects of the patients’ life. In our study we only observed distance between animals, while in humans the picture is more complex, hence comparisons need to be made carefully.

Humans with NCC also present with headaches, hydrocephalus and impaired memory and cognition (Garcia et al., 2003), hence further behavioural studies in animal are needed to assess these aspects in pigs.

For this study we were interested in studying pigs with NCC, hence only infected animals with more than three lingual cysts were selected. Further studies are needed to assess the behaviour of pigs with light infections, as this scenario can also be found in endemic areas. Moreover the number of cysticerci in the musculature of the infected pigs would be an aspect worth assessing, as this could further impact the behaviour of the animals.

Finally, the pig’s group affiliation should have been blinded to the reader; however this was not possible as the infected pigs had patterns that were clearly identifiable.

## 5 Conclusion

The results of the study indicated that NCC changed the behaviour of infected sows and had a negative effect on pig welfare. Sows with NCC spent less time at the feeding trough, were more passive and showed social isolation compared to non-infected control sows. The behavioural changes are indicative of decreased welfare. Infection should be avoided through provision of public health education to farmers, the animals should be diagnosed and treated and being a zoonotic disease cross-disciplinary control efforts should be initiated. Efforts to reinforce the animal welfare aspect are needed as this has so far been neglected.
