# Supplementary figures and images for: Disease behaviours of sows naturally infected with Taenia solium in Tanzania
Source: Vet Parasitol. 2017 Feb 15;235:69–74. doi: 10.1016/j.vetpar.2017.01.008 (PMC5331889; doi:10.1016/j.vetpar.2017.01.008)

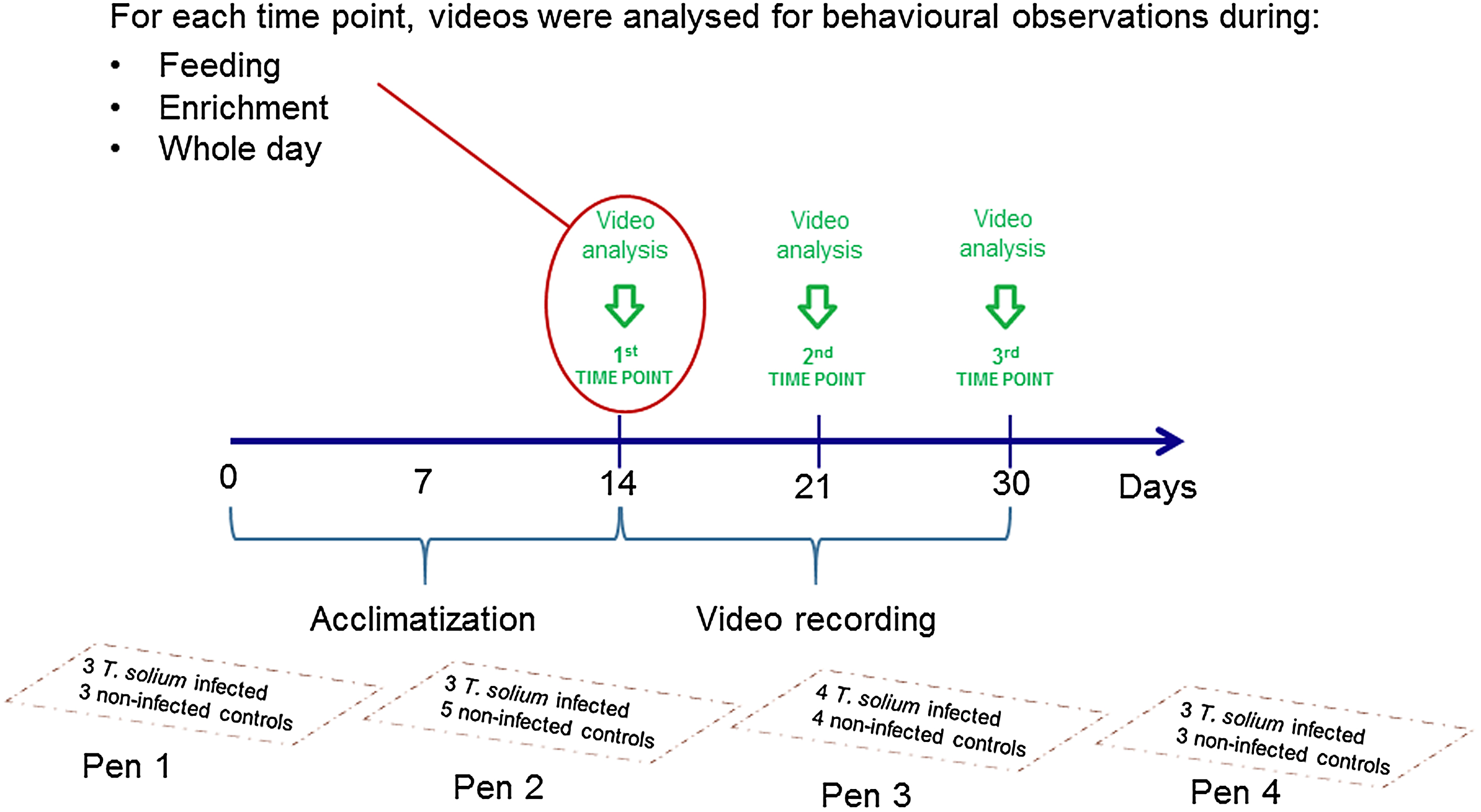

Supplement: Supplementary file 1 [file mmc1.jpg]
