# Supplementary material for: Disease behaviours of sows naturally infected with Taenia solium in Tanzania
Source: Vet Parasitol. 2017 Feb 15;235:69–74. doi: 10.1016/j.vetpar.2017.01.008 (PMC5331889; doi:10.1016/j.vetpar.2017.01.008)
Supplement: Supplementary file 2 [file mmc2.docx]

Table 1 adapted from Trevisan et al (2016): Number of brain cysts, pig age in months and presence or absence of seizures in 16 *T. solium* naturally infected pigs.

| **Pig**  **ID** | **No of brain**  **cysts** | **Pig age**  **(months)** | **Seizures** | **Included in**  **this study*** |
| --- | --- | --- | --- | --- |
| 1 | 247 | 36 | Yes | No |
| 2 | 241 | 36 | Yes | Yes |
| 3 | 47 | 18 | No | Yes |
| 4 | 418 | 18 | No | Yes |
| 5 | 4 | 24 | No | Yes |
| 6 | 178 | 12 | No | Yes |
| 7 | 4 | 24 | No | Yes |
| 8 | 88 | 12 | No | Yes |
| 9 | 27 | 12 | No | Yes |
| 10 | 21 | 18 | No | Yes |
| 11 | 36 | 8 | No | Yes |
| 12 | 90 | 12 | No | Yes |
| 13 | 10 | 8 | No | Yes |
| 14 | 117 | 18 | No | No |
| 15 | 41 | 18 | No | No |
| 16 | 136 | 18 | No | Yes |

*3 male pigs were not included in the study as these were kept in separate pens and were not part of the study design
